# New Appliances and Things Medical

**Published:** 1906-05-26

**Authors:** 


### NEW APPLIANCES AND THINGS MEDICAL.

### BIPSINE BREAD.

(The Bipsine Food Company, 39 Royal Park Terrace, Edinburgh.)

We have received a loaf of the above-named bread. The bread is of pleasant appearance, and possesses a good, palatable taste; in fact, it is quite as agreeable as good ordinary white bread, and appears to us to remain fresh longer. Even upon prolonged keeping it does not go bad, but simply becomes dry. The essential difference between Bipsine and ordinary white bread is that it is prepared differently, and that it contains thoroughly mixed with its substance a certain quantity of pepsine, and hence must be regarded as bearing somewhat the same relation to ordinary bread as peptonised milk does to ordinary milk. The bread is thus particularly suited to consumers who have difficulty in digesting ordinary bread; it is, in fact, according to the pamphlet accompanying it, "self-digesting," and requires less expenditure of energy upon the part of the consumer to digest it. We have not been able to examine the bread chemically, and do not know to what extent its proteid constituents have been actually changed by the added pepsine, or whether the advantages of it are confined to the help which will be accorded to its digestion in the stomach by the pepsine it actually contains. It can apparently be obtained from any baker, and therefore we assume that it is the flour from which the bread is made that is supplied by the Bipsine Food Company. According to the literature supplied, many subjects of weak digestion have received much benefit from the bread, and it certainly appears to us that it is worthy of an extensive trial at the hands of the medical profession.

### LIQUOR STROPHANTHI.

(Oppenheimer, Son, and Company, London.)

This well-known firm have placed upon the market a preparation of strophanthus seeds standardised to possess three times the strength of the B.P. tincture of strophanthus. The physiological action of strophanthus and its advantages over digitalis are now well known. One of the difficulties, however, in prescribing this remedy consists in the fact that the strophanthin of commerce is generally derived from an admixture of strophanthus seeds of different species, which, as a matter of fact, vary considerably in toxicity. Messrs. Oppenheimer, Sons, and Company have succeeded in obtaining the whole output of a definite variety of strophanthus seeds growing in a well-known strophanthus-producing area. It is these seeds of absolutely uniform origin which are exclusively used for making their liq. strophanthi, which is subsequently standardised to the above-mentioned strength. We think clinicians are much indebted to Messrs. Oppenheimer, Son, and Company for supplying them with a preparation of so important a drug of such constant strength, and we have no doubt that the profession will not be slow in taking advantage of it.

